# Supplementary material for: Chromothripsis during telomere crisis is independent of NHEJ, and consistent with a replicative origin
Source: Genome Res. 2019 May;29(5):737–49. doi: 10.1101/gr.240705.118 (PMC6499312; doi:10.1101/gr.240705.118)
Supplement: Supplemental Material [file supp_gr.240705.118_Supplemental_file_1.zip › contigs/annotated_contigs/DB115/contig.2.DB115_length_584_mean_cov_10.7174657534.docx]

**DB115_length_584_mean_cov_10.7174657534**

```
AAACGGGCTGCTTAACTAAGGAGGAATGAGGGAATCAACTGGTCGAGATAGTGTCTATCTAGAAATATCTGCATGACTGTGGTGAAGGG
 >chr16:79611211-79611524 + E=1e-176 p=1e-02
TCAATTGATACCTTAATTGCTTTGTGCCTGAGTCTCCTAATCTGTGTGAAGGAGATTAGAATATCTGCCTCTCTATTCATTGTTACAGG

TCAAAGATGGCATAGATCTGGTGTCCAGGAGACACTCGGTGCATGGTAGCTGTTGTCATCATAGCCCATGGAGGTTTTGGGTGACCTCC

AAAGGGCAGGGCTTCGGGAACATCACTGTCTTCTGCTCCACGGAAG|CAGAGAGTCACACCATGCTTTCTTCAGTATTAATGAATCCAA
                                                >chr16:79612221-79612492 + E=2e-151
AGGCTAAATGCCCAGGACTCAGGTGCATGCCGATATTTATATTGATACTCATGATCAATTTCTTCTCTACTTTCACACGATGTATCATT

TTTCAAATGTAAAGATTTTATTATGTAGTGCTTTGTTTCTAGATAGAATTCTATAGCTCTATTCATTTCTCATATTAACAGGATAATAT

TTAAATTTGAAAAAAACAGACATGGAAATATAAAATGAGAAAACTACAAGA
```
